# A public database to monitor the spread and impacts of high pathogenicity avian influenza viruses on albatrosses and petrels

**DOI:** 10.3897/BDJ.14.e186836

**Published:** 2026-05-15

**Authors:** Ralph E. T. Vanstreels, Patricia P. Serafini, Jolene Giacinti, Jane Younger, Kathryn P. Huyvaert, Michelle Wille, Laura Roberts, Amandine Gamble, Marcela M. Uhart

**Affiliations:** 1 ACAP High Pathogenicity H5Nx Avian Influenza Intersessional Correspondence Group, Hobart, Australia ACAP High Pathogenicity H5Nx Avian Influenza Intersessional Correspondence Group Hobart Australia; 2 Karen C. Drayer Wildlife Health Center, One Health Institute, University of California - Davis, Davis, United States of America Karen C. Drayer Wildlife Health Center, One Health Institute, University of California - Davis Davis United States of America; 3 Federal University of Santa Catarina, Florianópolis, Brazil Federal University of Santa Catarina Florianópolis Brazil; 4 ACAP Population and Conservation Status Working Group, Hobart, Australia ACAP Population and Conservation Status Working Group Hobart Australia; 5 Science and Technology Branch, Environment and Climate Change Canada, Winnipeg, Canada Science and Technology Branch, Environment and Climate Change Canada Winnipeg Canada; 6 Institute for Marine and Antarctic Studies, University of Tasmania, Hobart, Australia Institute for Marine and Antarctic Studies, University of Tasmania Hobart Australia; 7 College of Veterinary Medicine, Washington State University, Pullman, United States of America College of Veterinary Medicine, Washington State University Pullman United States of America; 8 WHO Collaborating Centre for Reference and Research, Peter Doherty Institute for Infection and Immunity, Melbourne, Australia WHO Collaborating Centre for Reference and Research, Peter Doherty Institute for Infection and Immunity Melbourne Australia; 9 Department of Microbiology and Immunology, University of Melbourne, Peter Doherty Institute for Infection and Immunity, Melbourne, Australia Department of Microbiology and Immunology, University of Melbourne, Peter Doherty Institute for Infection and Immunity Melbourne Australia; 10 Department of Agriculture, Western Cape Government, Elsenburg, South Africa Department of Agriculture, Western Cape Government Elsenburg South Africa; 11 Department of Public & Ecosystem Health, Cornell University, Ithaca, United States of America Department of Public & Ecosystem Health, Cornell University Ithaca United States of America

**Keywords:** disease, epidemiology, pathogen, Alphainfluenzavirus, Influenza A Virus, Orthomyxoviridae, bird, seabird, Diomedeidae, Hydrobatidae, Oceanitidae, Procellariidae, migratory species, wide-ranging species, threatened species, marine, global, Southern Ocean

## Abstract

**Background:**

High pathogenicity avian influenza (HPAI) viruses have rapidly emerged as a major global threat to wildlife, with severe consequences for seabird populations. Albatrosses and petrels (order Procellariiformes) are particularly vulnerable due to their long lifespan, low reproductive rates and strong site fidelity. Since 2021, HPAI viruses have caused unprecedented mortality in seabird communities worldwide and have expanded into the core range of procellariiform species, including sub-Antarctic and Antarctic regions.

**New information:**

In response to the urgent need for timely, species-relevant information, the Agreement on the Conservation of Albatrosses and Petrels (ACAP) established the High Pathogenicity H5Nx Avian Influenza Intersessional Correspondence Group (HPAI-ICG), which developed the ACAP HPAI database — an openly accessible, regularly updated resource that consolidates all known suspected and confirmed HPAI events involving procellariiform birds. The database compiles information from global and national reporting systems, scientific literature, genetic repositories, government communications and direct expert notifications. Events are standardised using transparent case definitions, cross-referenced and validated by subject-matter experts and complemented by additional data on case impacts and viral characteristics. The database provides a critical decision-support tool for governments, researchers, conservation practitioners and tourism operators, contributing to the planning and implementation of HPAI biosafety, surveillance, monitoring and outbreak response activities.

## Introduction

Albatrosses and large petrels are amongst the most threatened groups of birds in the world, with nearly 70% of species considered at risk of extinction due to a combination of threats including bycatch in fisheries, invasive species, marine pollution, climate change and disease (Phillips et al. 2016, Dias et al. 2019). Due to their long lifespan, slow reproductive rate and strong site fidelity, these birds are particularly vulnerable to adult mortality and habitat disruption (Phillips et al. 2016). The Agreement on the Conservation of Albatrosses and Petrels (ACAP) provides a framework for international cooperation to protect these seabirds through population monitoring, threat mitigation and habitat protection (Cooper et al. 2006). ACAP currently lists 31 species, including all albatrosses (Diomedeidae, 22 spp.) and some petrels and shearwaters (Procellariidae, 9 spp.), reflecting their conservation status and the need for multilateral action to address conservation threats (Agreement on the Conservation of Albatrosses and Petrels 2020). In addition to the ACAP-listed species, the order Procellariiformes comprises a further 117 species, including petrels, shearwaters, prions and fulmars (Procellariidae, 89 spp.), northern storm-petrels (Hydrobatidae, 18 spp.) and southern storm-petrels (Oceanitidae, 10 spp.) (Gill et al. 2025).

Since 2021, high pathogenicity avian influenza (HPAI) viruses of the subtype H5 have emerged as a global threat to the conservation of wild birds (Klaassen and Wille 2023). The destructive potential of these viruses on seabird communities was first described in Europe, Africa and North America, where outbreaks in 2021–2022 decimated tens of thousands of seabirds (Abolnik et al. 2023, Avery‐Gomm et al. 2024, Knief et al. 2024, Lane et al. 2024, Tremlett et al. 2024). In October 2022, the viruses spread to South America for the first time, leading to mortality of over 500,000 seabirds and 50,000 marine mammals (Uhart et al. 2024, Kuiken et al. 2025, Uhart and Vanstreels 2025). From South America, HPAI viruses spread to sub-Antarctic islands in the Southwest Atlantic Ocean in October 2023 (Banyard et al. 2024) and subsequently to the Antarctic Peninsula in January 2024 (Aguado et al. 2024, Ogrzewalska et al. 2025). Beginning in September 2024, there was a further range expansion to sub-Antarctic islands in the South Atlantic Ridge and South Indian Ocean (Clessin et al. 2025, Steinfurth et al. 2025).

The impact of HPAI viruses has been catastrophic for some seabird populations. In the Northern Hemisphere, skuas, terns and gannets experienced particularly severe impacts, experiencing population decreases up to 30–70% (Knief et al. 2024, Lane et al. 2024, Tremlett et al. 2024). However, not all seabird groups were similarly impacted and, in the case of procellariiform birds, there were no HPAI-related mortality events in the Northern Hemisphere apart from small mortality clusters of northern fulmars (*Fulmarus glacialis*) in Canada and the Netherlands (Caliendo et al. 2025, Canadian Wildlife Health Cooperative 2025). This is not unexpected, given that the abundance and diversity of procellariiform birds are largely concentrated in the Southern Hemisphere, especially between 37°S and 59°S (Chown et al. 1998). Consequently, the spread of HPAI viruses to the sub-Antarctic Region in October 2023 was recognised as a major escalation in the conservation risk to this seabird group due to increased exposure probability. This risk materialised three months later, in January 2024, when mass mortalities of albatrosses due to HPAI were reported at the Falkland Islands (Islas Malvinas) and South Georgia (Islas Georgias del Sur) (Bennison et al. 2024, Kuepfer and Stanworth 2024).

The ACAP High Pathogenicity H5Nx Avian Influenza Intersessional Correspondence Group (HPAI-ICG) was established in May 2023 in response to the global crisis caused by HPAI viruses. The HPAI-ICG aims to compile up-to-date information, continually assess the risk and communicate and provide guidance to decision-makers and stakeholders within ACAP. Importantly, the HPAI-ICG focuses on prevention and risk mitigation, with provisions to equip ACAP members and scientists for early detection and response. Amongst other activities, this group has organised in-person biosafety training workshops and published a variety of HPAI-related materials, such as guidelines for biosafety, preparedness and response to outbreaks (Serafini et al. 2024), training videos on the use of personal protective equipment and alert posters for crew on board fishing vessels (available at https://acap.aq/resources/disease-threats/avian-flu).

As HPAI is listed as a notifiable disease by the World Organisation for Animal Health (WOAH), information about HPAI detections is publicly available through the World Animal Health Information System (WAHIS). However, reporting — particularly for wildlife — can be delayed, incomplete or inconsistent across countries. For example, many notifications from the Antarctic Region are not included in WAHIS as researchers undertaking the testing are not the national reference laboratory and are, therefore, unable to make the notification. Moreover, given the remoteness of events, confirmatory testing at the reference laboratory can be delayed by months. Another problem is that although there are public databases about HPAI detections, their interfaces may be specifically designed to special-interest audiences, such as the poultry industry, animal health authorities, molecular biologists etc. As a result, extracting information specific to procellariiform birds from these sources may require user registration and authorisation (e.g. GISAID), downloading spreadsheets and searching through raw data (e.g. governmental databases), multi-step searches in online database interfaces (e.g. WAHIS) or consultation of the scientific literature. In addition, relevant information may be in a variety of languages, with unique local names being used to refer to procellariiform species. Moreover, because these sources of information can contain errors or may overlap with one another, consolidating their data also requires careful cross-referencing to identify matching records and resolve discrepancies.

These limitations can result in gaps and delays in situational awareness that can hamper conservation efforts and the governance of activities that directly or indirectly involve procellariiform birds (research, disease surveillance, tourism, fisheries etc.). As the HPAI situation continues to unfold, the HPAI-ICG has developed the ACAP HPAI database to systematise and publicise information about all known suspected and confirmed HPAI cases in procellariiform birds.

## General description

### Purpose

This database aims to provide consolidated and up-to-date information about HPAI events affecting albatrosses, petrels and other procellariiform birds worldwide. While important global and national reporting systems exist, they were designed for broad animal health purposes and may not always provide the species-level detail or contextual metadata most relevant to the protection of albatrosses and petrels. The ACAP HPAI database helps bring together diverse sources of information and expert notifications into a single, regularly updated resource that is straightforward to access and interpret — while complementing, not replacing, official reports by countries to WOAH and other organisations. The database is primarily targeted at those involved in seabird research and conservation, including government officials of ACAP parties, researchers, conservation managers, fisheries, tourism operators, bird watchers, amongst others. Access to regularly updated information on the spread and impacts of HPAI on procellariiform birds may be particularly important to provide information for the implementation and scaling of biosafety measures, the prioritisation of research, population monitoring and disease surveillance activities and the implementation of outbreak mitigation strategies.

### Additional information

This database adopts FAIR (Findable, Accessible, Interoperable and Reusable) and CARE (Collective Benefit, Authority, Responsibility and Ethics) principles (Sterner and Elliott 2024), aiming to provide open, interoperable access to HPAI detections while respecting data ownership, national sovereignty and the sensitivities of reporting outbreaks in threatened species, while promoting trust and shared stewardship amongst data contributors and users. Although the creation of this database was primarily motivated by the global emergence HPAI viruses of the subtype H5 since 2020 (i.e. Goose/Guangdong viruses of the subclade 2.3.4.4b), cases involving other HPAI subtypes or lineages may be included in the future.

## Sampling methods

### Sampling description

Information was compiled from both public sources and direct notifications to the ACAP HPAI-ICG. Raw data were downloaded from international and national databases of animal health and genetic information (Table 1). Other sources, such as press releases by national animal health and conservation/environmental authorities, official governmental websites, research reports, scientific papers and preprints and conference presentations were also collated.

Data from international and national databases were pre-filtered using R (R Core Team 2023) and *tidyverse* (Wickham 2023) to select rows containing one or more keywords indicative of procellariiform birds (Suppl. material 1). The compiled information was then assessed by an expert (R.E.T. Vanstreels), who checked and cross-referenced the information and consolidated it into a database of HPAI events. An HPAI event is defined as an instance where one or more individuals of a given procellariiform species were confirmed or suspected to have been infected by an HPAI virus at a given location and date. The consolidated database of HPAI events was reviewed and validated by a panel of experts (ACAP HPAI-ICG members). When necessary, the original sources of information (researchers, government officers etc.) were contacted to supplement incomplete records or to resolve any metadata inconsistencies amongst different sources.

Based on the critical assessment of the best-available information about field observations and diagnostic methods employed, each HPAI event was classified as either “suspected” or “confirmed” following the case definitions presented in Table 2. In these definitions, the expression “suspected morbidity or mortality event” is used to refer to above-background level of mortality and/or the observation of individuals presenting with one or more of the following clinical signs or post-mortem findings: sudden death, atypical behaviour (loss of fear, landing in unusual places, walking or swimming in circles etc.), loss of coordination or balance, inability to walk or stand, repetitive movements (sideways neck shaking, pedalling or wing flapping etc.), head and neck tremors, torticollis/opisthotonus, ocular discharge, periorbital oedema, oedema and opacification of the cornea or nictitating membrane, darkening of the iris, nasal discharge, sneezing, coughing, difficulty breathing, diarrhoea, oedema and necrotic or haemorrhagic lesions on feet, congestion or haemorrhage in lungs or necrotic or haemorrhagic lesions in the pancreas.

## Geographic coverage

### Description

**Figure 1.**
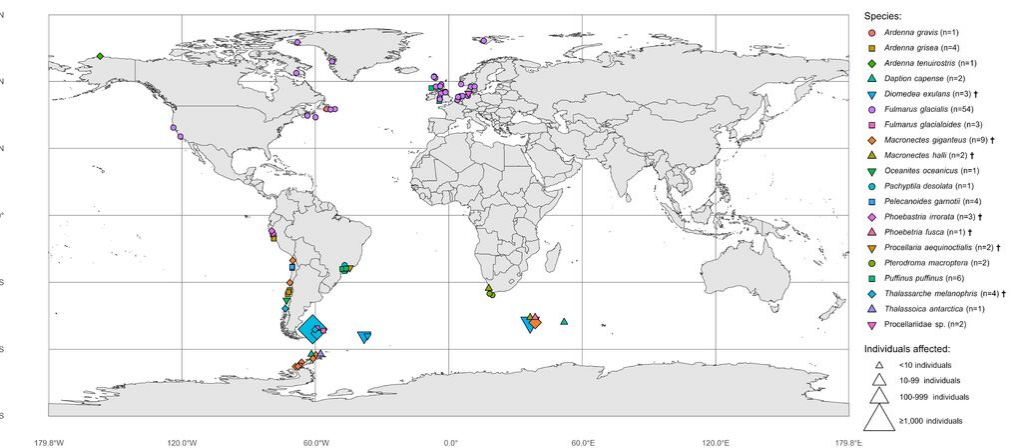
Geographic distribution of confirmed HPAI events in procellariiform birds. HPAI events are with coloured symbols by species (shape and colour) and number of individuals affected (size). The total number of confirmed HPAI events recorded for each species is indicated in the legend (n). Dagger symbols (†) indicate species listed in Annex 1 of the Agreement on the Conservation of Albatrosses and Petrels (ACAP). Data updated as of 31 December 2025.

The database provides global coverage of data on documented suspected or confirmed cases of HPAI in Procellariiformes birds. The nomenclature of countries/territories was standardised following the United Nations (United Nations Statistics Division 2025). Fig. 1 provides an overview of the overall distribution of confirmed HPAI events by host species and number of individuals affected.

## Taxonomic coverage

### Description

The database covers all detections of HPAI viruses in procellariiform birds. HPAI viruses comprise any *Alphainfluenzavirus influenzae* determined as high pathogenicity in accordance with the WOAH Terrestrial Manual (https://www.woah.org/en/what-we-do/standards/codes-and-manuals/). Procellariiform birds are defined following the International Ornithologists' Union (Gill et al. 2025). Table 3 provides an overview of the number of HPAI events and individuals affected by host species and event category.

### Taxa included

**Table:** 

Rank	Scientific Name	
species	Alphainfluenzavirus influenzae	
order	Procellariiformes	

## Traits coverage

The following variables were recorded for each HPAI event:

1. Morbimortality: Whether the HPAI event was associated with ill or deceased individuals (TRUE) or it corresponds to HPAI virus detection in live asymptomatic individuals (FALSE);
2. Individuals: The number of individuals affected in each HPAI event refers to the number of birds of that species that were ill or deceased or the number of live asymptomatic individuals in which HPAI viruses were detected. When no information was provided in the original report, it was presumed to be 1;
3. Estimate method: Method through which the number of individuals affected in the event was estimated. The following categories are possible: a) “Direct count”, when the number of individuals affected was directly counted; b) “Field estimate”, when the number of individuals affected was not directly counted, but was estimated through field surveys (e.g. counting carcasses in some sub-areas then extrapolating to the total affected area); and c) “Indirect estimate”, when the number of individuals affected was extrapolated from population data (e.g. estimation of above-background mortality, based on long-term population monitoring data);
4. Category and subcategory: Category according to the case definitions (see Table 2);
5. Subtype: Subtype of the haemagglutinin (H) and neuraminidase (N) viral antigens, if known. “Hx” and “Nx” are used to represent undetermined subtypes;
6. Isolates: Name of the virus isolate(s) associated with the event, if any, for which there is genomic sequence data. Isolate names follow the standard of the World Health Organization: "Virus type / Host species / Geographical origin / Strain number / Year of isolation". Incorrect information or typos are maintained as in the original;
7. Comments: Additional information about the context in which the affected individuals were found, clinical signs, diagnostic methods, methods used to estimate morbidity/mortality, discrepancies in information provided by different sources etc. when available;
8. Source: Sources of information for the HPAI event. When referring to information from public databases, the corresponding accession codes are provided (“outbreakId" for WAHIS, "Isolate_Id" for GISAID etc.). The complete references and links for the sources are provided in a secondary dataset (“Sources”).

## Temporal coverage

**Data range:** 2021-10-15 – 2025-12-31.

### Notes

**Figure 2.**
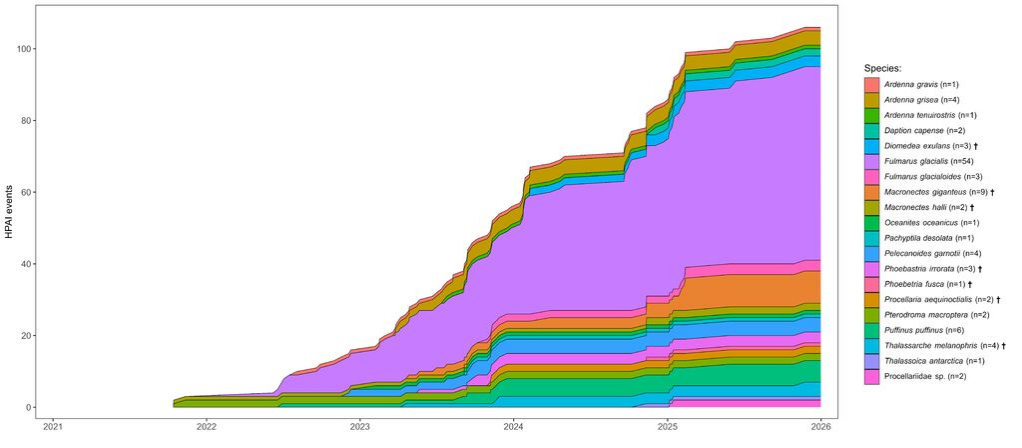
Temporal distribution of confirmed HPAI events in procellariiform birds. HPAI events are represented with cumulatively stacked areas by species (colour). The total number of confirmed HPAI events recorded for each species is indicated in the legend (n). Dagger symbols (†) indicate species listed in Annex 1 of the Agreement on the Conservation of Albatrosses and Petrels (ACAP). Data updated as of 31 December 2025.

The database comprises all HPAI events up to 31 December 2025, with the earliest known HPAI event dating back to 15 October 2021. The database will continue to be updated after release on a monthly basis or whenever new HPAI events are brought to the attention of the ACAP HPAI-ICG. Fig. 2 provides an overview of the temporal distribution of recorded HPAI events by host species.

## Usage licence

### Usage licence

Other

### IP rights notes

Records are released under the Creative Commons Attribution 4.0 International licence (CC‑BY-4.0).

## Data resources

### Data package title

ACAP HPAI database

### Resource link

https://doi.org/10.5281/zenodo.17683269

### Number of data sets

3

### Data set 1.

#### Data set name

ACAP HPAI Events

#### Data format

Tab-Separated Values (TSV)

#### Description

This dataset assembles global information about HPAI events, i.e. instances where high pathogenicity avian influenza (HPAI) viruses were demonstrated or are believed to have occurred, in procellariiform birds (albatrosses, petrels, shearwaters, prions, fulmars and storm-petrels). In addition to basic information about the event category ("suspected" or "confirmed" as per case definitions), date, location (including both text descriptors and geographical coordinates) and procellariiform species involved, supporting information about case impacts (morbimortality, number of individuals affected, method used to estimate the number of individuals affected) and viral characteristics (antigenic subtype and availability of genomic data) associated with each HPAI event are summarised.

**Data set 1.** 

Column label	Column description
AccessionCode	Accession code that is unique to each event. Numbers are assigned by combining the date (yyyymmdd format), an underscore symbol and a two-digit integer (assigned sequentially by order of inclusion in the database).
Date	Date (yyyy-mm-dd format) when the event was recorded.
Region	Region or continent where the event was recorded.
Location1	Upper level descriptor (country, territory, archipelago etc.) of the event location.
Location2	Mid-level descriptor (state, province, island etc.) of the event location.
Location3	Lower level descriptor (municipality, settlement, colony, beach etc.) of the event location.
Latitude	Latitude (decimal degrees, datum WGS84) of the event location.
Longitude	Longitude (decimal degrees, datum WGS84) of the event location.
Species	Species Latin name. When the species could not be determined, the lowest taxonomical level is used instead.
EnglishName	Species common name in English.
SpanishName	Species common name in Spanish.
FrenchName	Species common name in French.
Family	Taxonomic family of the procellariiform species.
IUCN	Abbreviated conservation status of the procellariiform species as listed by the International Union for Conservation of Nature Red List (https://www.iucnredlist.org/).
ACAP	Whether or not the procellariiform species is listed in Annex 1 of the Agreement on the Conservation of Albatrosses and Petrels (https://acap.aq/resources/acap-species/).
Morbimortality	Whether the event was associated with illness or death (TRUE) or it represented virus detection on samples from apparently healthy individuals (FALSE).
Individuals	Number of individuals in which the virus was detected or that are known/estimated to have become ill or died in association with the event.
EstimateMethod	Method through which the number of individuals affected in the event was estimated: “Direct count” (number of individuals was directly counted in the field), “Field estimate” (number of individuals was estimated through field surveys) or “Indirect estimate” (number of individuals was extrapolated from population census data).
Category	Category of the event (Suspected or Confirmed) according to the case definitions.
Subcategory	Subcategory of the event (S1, S2, S3 etc.) according to the case definitions.
Subtype	Subtype of the haemagglutinin (H) and neuraminidase (N) viral antigens.
Isolates	Name of the virus isolate(s) associated with the event.
Comments	Additional information about the context in which the affected individuals were found, clinical signs, diagnostic methods, methods used to estimate morbidity/mortality, discrepancies in information provided by different sources etc.
Source	Sources of information for the event.

### Data set 2.

#### Data set name

ACAP HPAI Sources

#### Data format

Tab-Separated Values (TSV)

#### Description

This dataset provide full references for the source material listed in the “Source” column of the “ACAP HPAI Events” dataset.

**Data set 2.** 

Column label	Column description
SourceCited	Short name of the source of information as featured in the “Sources” column of the “Events” dataset.
Reference	Reference in Harvard format or detailed text description of the source of information.
URL	Uniform Resource Locator (URL) for the source material. Digital Object Identifier (DOI) links are preferred when available.

### Data set 3.

#### Data set name

ACAP HPAI Update History

#### Data format

Tab-Separated Values (TSV)

#### Description

This dataset provides a chronological account of the changes and corrections made to the “ACAP HPAI Events” and “ACAP HPAI Sources” datasets, ensuring data transparency and traceability.

**Data set 3.** 

Column label	Column description
UpdateDate	Date (yyyy-mm-dd format) when the database update was released.
Description	Description of changes made, including the accession codes of HPAI events that were added or modified in each update.

## Supplementary Material

51A08155-CD17-53DA-B583-29DBD25B586C 10.3897/BDJ.14.e186836.suppl1 Supplementary material 1 Keyword pre-filtering script Data type R script Brief description Example of R script used to automate the screening of large public HPAI datasets to extract the subset of records that may be related to procellariiform birds. File: oo_1516136.R https://binary.pensoft.net/file/1516136 Vanstreels RET, Serafini PP, Giacinti J, Younger J, Huyvaert KP, Wille M, Roberts L, Gamble A, Uhart MM

## Figures and Tables

**Table 1.** Public databases used to compile information about potential HPAI cases in procellariiform birds.

**Database**	**URL**
GenBank	https://www.ncbi.nlm.nih.gov/genbank/
Global Animal Disease Information System (EMPRES-i+)	https://empres-i.apps.fao.org/diseases
Global Initiative on Sharing All Influenza Data (GISAID)	https://gisaid.org/
Scientific Committee for Antarctic Research (SCAR)	https://scar.org/library-data/avian-flu
World Animal Health Information System (WAHIS)	https://wahis.woah.org/
Animal and Plant Health Agency (APHA)	https://www.gov.uk/government/publications/avian-influenza-in-wild-birds
Animal and Plant Health Inspection Service (APHIS)	https://www.aphis.usda.gov/livestock-poultry-disease/avian/avian-influenza/hpai-detections/wild-birds
Centro Nacional de Epidemiología, Prevención y Control de Enfermedades (CDC Perú)	https://www.dge.gob.pe/sala-influenza-aviar/SITUACION-AH5.html
Falkland Islands Department of Agriculture (FIDA)	https://www.falklands.gov.fk/agriculture/avian-influenza
Interagency Surveillance Program for Avian Influenza Viruses in Wildlife (ISPAIVW)	https://cfia-ncr.maps.arcgis.com/apps/dashboards/89c779e98cdf492c899df23e1c38fdbc
Ministério da Agricultura e Pecuária (MAPA)	https://mapa-indicadores.agricultura.gov.br/publico/extensions/SRN/SRN.html
Servicio Agrícola y Ganadero (SAG)	https://www.sag.gob.cl/ambitos-de-accion/influenza-aviar-ia
Wildlife Health Information Sharing Partnership (WHISPers)	https://whispers.usgs.gov/home

**Table 2.** Case definitions for HPAI events in procellariiform birds.

**Category**	**Subcategory**	**Criteria**
Suspected	S1	Suspected morbidity or mortality event was noted; no diagnostic test results are available to date.
	S2	Suspected morbidity or mortality event was associated with a positive result in influenza A virus (IAV) of any subtype in rapid antigen testing (direct immunoassay targeting IAV antigen).
	S3	Suspected morbidity or mortality event was associated with a positive result for IAV of unknown subtype in molecular testing or virus isolation.
	S4	Suspected morbidity or mortality event was associated with a positive result for IAV of subtype H5 or H7 in serological testing (haemagglutination inhibition test).
	S5	In the absence of a suspected morbidity or mortality event, a positive result was obtained for IAV of subtype H5 or H7 in molecular testing or virus isolation.
	S6	Suspected morbidity or mortality event was associated with a positive result for IAV of subtype H5 or H7 in molecular testing or virus isolation.
	+	This indicator is added to subcategories S1 to S5 when there were one or more confirmed HPAI detections (as per criteria below) in another animal species at the site (or within a 500 km radius) within a 3-month period before or after the suspected event.
Confirmed	C1	A positive result was obtained for IAV in molecular testing using primers/probes specifically designed and validated for the detection of HPAI strains (e.g. primers/probes specific to Goose/Guangdong clade 2.3.4.4b strains) at a national reference laboratory.
	C2	A positive result was obtained for IAV of subtype H5 or H7 in molecular testing or virus isolation and the strain was confirmed to be HPAI through genome sequencing (specifically, of the haemagglutinin cleavage site) or determination of the intravenous pathogenicity index.

**Table 3.** Overview of the distribution of the number of HPAI events and individuals affected in procellariiform birds, by event category (confirmed or suspected) and species. Data updated as of 31 December 2025.

**Species**	**English common name**	**Number of HPAI events**		**Number of individuals affected**	
		**Confirmed**	**Suspected**	**Confirmed**	**Suspected**
*Ardenna gravis*	Great Shearwater	1	1	1	1
*Ardenna grisea*	Sooty Shearwater	4	0	9	0
*Ardenna tenuirostris*	Short-tailed Shearwater	1	0	3	0
*Daption capense*	Pintado Petrel	2	0	2	0
*Diomedea exulans*	Wandering Albatross	3	2	209	3
*Fulmarus glacialis*	Northern Fulmar	54	0	77	0
*Fulmarus glacialoides*	Southern Fulmar	3	0	3	0
*Macronectes giganteus*	Southern Giant Petrel	2	0	5	0
*Macronectes halli*	Northern Giant Petrel	9	0	29	0
*Oceanites oceanicus*	Wilson's Storm Petrel	1	0	1	0
*Pachyptila desolata*	Antarctic Prion	1	0	1	0
*Pelecanoides garnotii*	Peruvian Diving Petrel	4	0	4	0
*Phoebastria irrorata*	Waved Albatross	3	0	6	0
*Phoebetria fusca*	Sooty Albatross	1	0	5	0
*Procellaria aequinoctialis*	White-chinned Petrel	2	0	2	0
*Pterodroma macroptera*	Great-winged Petrel	2	0	2	0
*Puffinus puffinus*	Manx Shearwater	6	0	6	0
*Thalassarche melanophris*	Black-browed Albatross	4	1	53042	500
*Thalassoica antarctica*	Antarctic Petrel	1	0	1	0
Procellariidae	Unidentified Petrel	2	1	2	1
Total		106	5	53410	505
